# Adapted Linear Binning Method to Assess Pulmonary Ventilation and Perfusion in Children Using Lung Matrix Pencil Decomposition (MP)‐MRI

**DOI:** 10.1002/mrm.70544

**Published:** 2026-08-13

**Authors:** Marion Curdy, Carmen Streibel, Oliver Bieri, Elisabeth Kieninger, Florian Wyler, Lukas Ebner, Grzegorz Bauman, Philipp Latzin

**Affiliations:** ^1^ Pediatric Respiratory Medicine, Department of Pediatrics Inselspital, Bern University Hospital, University of Bern Bern Switzerland; ^2^ Graduate School for Health Sciences University of Bern Bern Switzerland; ^3^ Division of Radiological Physics, Department of Radiology University of Basel Hospital Basel Switzerland; ^4^ Department of Biomedical Engineering University of Basel Allschwil Switzerland; ^5^ Department of Radiology and Nuclear Medicine Lucerne Cantonal Hospital, University of Lucerne Lucerne Switzerland; ^6^ Department of Diagnostic, Interventional and Paediatric Radiology Inselspital, Bern University Hospital, University of Bern Bern Switzerland

**Keywords:** defect percentage, linear binning, lung, MP‐MRI, pediatrics, ventilation and perfusion

## Abstract

**Purpose:**

To adapt and validate a linear binning technique, developed for hyper‐polarized 129Xe MRI, for functional lung MRI with matrix‐pencil decomposition (MP)‐MRI.

**Methods:**

First, a dedicated normalization was applied to the perfusion‐weighted and the ventilation‐weighted map histograms. Then, using 154 MP‐MRI scans of healthy children, reference bins were defined around the peak of the averaged histogram as normal (±1 SD), high (> 1 SD), low (between −1 SD and −2 SD), and defect (< −2 SD), and subsequently applied to a validation dataset comprising healthy children (HC, *N* = 41) and children with cystic fibrosis (CF, *N* = 30) to evaluate the accuracy of the classification and its discriminatory power. Furthermore, a third dataset comprising children with CF pre and post elexacaftor/tezacaftor/ivacaftor (ETI) therapy (*N* = 24) was binned to evaluate the method's sensitivity to treatment effects. Standard outcome parameters, computed with a median threshold, served as a comparison.

**Results:**

The adapted linear binning resulted in significantly higher defect and low percentages for perfusion and ventilation between children with CF and HC (*p* values < 0.0001) with high discrimination (AUCs > 0.85). This was comparable to the standard median‐threshold method. Two illustrative cases were included to demonstrate the complementary granularity of the linear binning method. Although standard thresholding indicated improvement in both ventilation and perfusion defects pre and post ETI, linear binning showed that perfusion improvement was restricted to the low category.

**Conclusion:**

A linear binning method for MP‐MRI was developed and validated, providing a healthy reference based on a large dataset and advancing functional lung image processing.

## 1 Introduction

Functional lung MRI has proven to be an effective technique for longitudinal monitoring of lung function [1, 2, 3]. In pediatric settings, functional imaging techniques such as self‐gated non‐contrast‐enhanced (SENCEFUL) [4], phase resolved functional lung imaging (PREFUL) [5], and matrix pencil decomposition [6] (MP) are advantageous. They allow children to breathe freely during the scan without requiring the use of contrast agents or inhaled gaseous tracers. Furthermore, they share the key advantage of generating both ventilation‐weighted and perfusion‐weighted maps simultaneously. These non‐contrast, free breathing MRI techniques have all demonstrated promising results for lung function monitoring in pediatric settings [3, 7, 8, 9, 10, 11, 12, 13, 14].

Our center uses MP‐MRI, a derivative of Fourier decomposition more robust against truncation artifacts from short‐time series [6]. The Fourier decomposition is a highly reproducible technique [15, 16], giving results comparable with other functional lung MRI techniques such as hyperpolarized (HP) ^129^Xe MRI and dynamic contrast‐enhanced perfusion MRI in humans and large animals [17, 18, 19]. MP‐MRI has been shown to be repeatable in healthy children as well as in children with cystic fibrosis (CF) [20], and MP‐MRI outcomes for both ventilation and perfusion have been shown to correlate with lung function in children with CF and congenital diaphragmatic hernia [7, 20, 21, 22, 23].

The standard outcome variables of MP‐MRI, and several functional imaging techniques, are ventilation and perfusion defect percentages (VDP/QDP respectively). They represent the proportion of the lung parenchyma with impaired function [24]. Since MRI lacks an absolute scale, functional MRI studies often use a threshold‐based approach to compute these outcomes, for example, the 70% of the median [25]. This intra‐individual, slice‐dependent method computes a threshold for each lung slice separately, and all voxels below this threshold are classified as a defect. However, this method has some limitations. It is mathematically bounded; the defect percentage cannot reach 50% and above. It is also highly dependent on slice variations; in case of homogeneous impairment, the method might underestimate the true defect percentage. This binary outcome also does not accurately reflect the heterogeneous underlying physiology of the lungs [26]. Several algorithms have been developed for HP MRI to classify the ventilation in different categories, such as the linear binning [27, 28] and the (adaptive) k‐means [29, 30]. These algorithms result in categories such as defect and low ventilation/perfusion, and provide additional information that could be combined with the standard VDP/QDP outcome.

A linear binning approach was developed for HP ^129^Xe MRI and has been successfully applied to both healthy populations and to patients with pulmonary diseases such as CF and asthma [27, 31, 32, 33, 34]. It has proven to be more effective than thresholding methods in detecting even subtle low‐ventilated regions in patients with milder diseases like asthma [31] and age‐related changes [27]. According to its authors, linear binning has several advantages: (i) it bins the pulmonary ventilation distribution in four categories: normal, high, low, and defect instead of a binary outcome, (ii) the thresholds refer to a fixed healthy reference, not being sensitive to intra‐individual variations, and (iii) it relies on fewer assumptions than the k‐means algorithm while being easier to understand and to relate to physiology [27].

Generally, HP ^129^Xe and proton‐based lung MRI provide fundamentally different signal intensities in the lung. A key feature of MP‐MRI, however, is its robust, simultaneous extraction of both regional ventilation and perfusion. Perfusion‐weighted maps are strongly right‐skewed due to high‐intensity signals from the vasculature. In contrast, ventilation‐weighted maps are more homogeneous, with fewer high‐intensity voxels, reflecting the more limited signal range from parenchymal tissue compression during respiratory movement. Thus, MP‐MRI images call for an adaptation of the original linear binning method. Consequently, while the method has been adapted for PREFUL ventilation‐weighted maps [35, 36], it has not yet been applied to perfusion‐weighted maps or to data from MP‐MRI.

This article describes the derivation and validation of a linear binning method adapted to MP‐MRI images. We first use a large external healthy population to derive fixed reference thresholds to be applied for linear binning of individual scans. We then validate the adapted linear binning method by applying it to a validation dataset comprising children with CF and healthy ones. Last, we use individual cases as well as group mean treatment effects in children with CF to illustrate its potential usefulness as an application of real‐world data.

## 2 Methods

### 2.1 Study Design

This study was an exploratory retrospective analysis of data collected from previous studies conducted in our laboratory [8, 37, 38]. The studies were approved by the Ethics Committee of Bern, Switzerland (ID 2019‐01591, 2017‐00279, 2019‐01072), and each participant or legal guardian gave written informed consent. This study was divided into two main parts, as follows: an overview of the datasets can be seen in Figure S1.

In the first part, the HP ^129^Xe linear binning method was adapted to MP‐MRI and validated against the standard median‐based thresholding. The reference linear binning thresholds computed with a healthy reference dataset of randomly chosen children from the Bern‐Basel Infant Lung Development cohort [37] (*N* = 154, ˜80% of the data) were used. This defined the reference bins (defect, low, normal and high), used on any other dataset.

The discriminability of the adapted linear binning was validated with a dataset comprising the remaining 20% of healthy children in the Bern‐Basel Infant Lung Development cohort (*N* = 41) as well as children with CF from a previously published study [38] (*N* = 30). This discriminative power was validated against that achieved with the standard median thresholding [25].

In the second part, an application of the linear binning was tested with an already published dataset comprising 24 children with CF under elexacaftor/tezacaftor/ivacaftor (ETI) therapy [8], who had an MRI before the start of their therapy (average in months: 8.75 ± 4.69) as well as an MRI on average 4.70 (±2.59) months after ETI initiation. An individual case analysis of two patients pre‐ETI treatment is presented to illustrate the potential usefulness of the linear binning. The two children were chosen to exemplify scenarios where the map‐dependent median threshold and the reference‐based linear binning yield divergent classifications of lung function.

The use of the adapted linear binning method to identify longitudinal treatment effects was in turn evaluated. The improvements of the defect and low‐function areas pre and post ETI therapy were compared to the results obtained with the standard median thresholding.

### 2.2 Functional Lung Imaging

MRI examinations were conducted using a clinical 1.5T whole‐body scanner (MAGNETOM Aera, Siemens Healthineers, Erlangen, Germany). Functional scans were performed in supine position during free breathing with a multi‐slice 2D time‐resolved ultra‐fast balanced steady‐state free precession (uf‐bSSFP) pulse sequence [39]. Approximately 8–14 coronal slices were acquired to cover the whole chest. The full process of the acquisition and data image post‐processing by TrueLung is described in detail elsewhere [25]. All acquired images were processed with the MP algorithm [6] to create the ventilation‐ and perfusion‐weighted lung parenchyma maps. All final lung parenchyma masks generated by the algorithm were visually inspected by an experienced reader and manually corrected if necessary. The vasculature signal was identified on the extracted perfusion‐maps, where static or non‐pulsating structures such as mucus plugs, bronchiectasis or inflammatory consolidations are not generating a high signal. Voxels were classified as vasculature if their signal was above the 90th percentile, and the vasculature signal was removed from each perfusion‐weighted and ventilation‐weighted map.

### 2.3 Linear Binning—Reference

In contrast to the median‐based thresholding that is performed individually for every perfusion‐ and ventilation‐weighted image, the linear binning relies on thresholds that are derived from a large group of healthy controls, see Figure 1. This method first builds a healthy reference distribution from which the thresholds for the defect, low, normal, and high bins are defined mathematically. To this end, all images are first normalized individually using a defined cut‐off value, with all voxel values exceeding the cut‐off set to one. Then, all normalized images are stacked together in a reference histogram.

**FIGURE 1:**
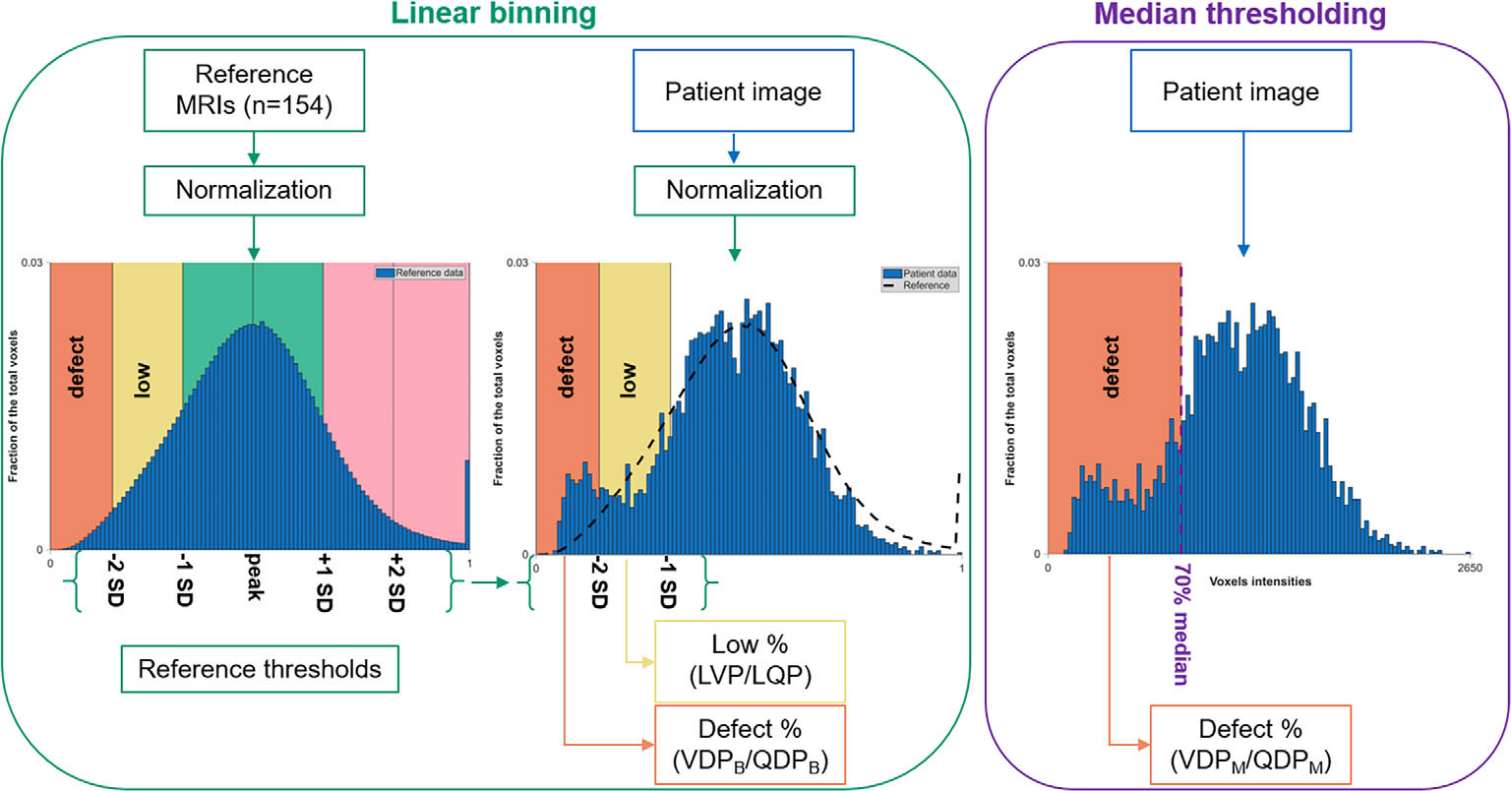
Methodological differences between the linear binning method and the median thresholding. The linear binning panel describes how the method functions. Initially, a reference is built by normalizing each map and stacking the values into one big distribution. The bins are then defined around the peak of the distributions, with the low and the defect bin being the peak −1 SD and −2 SD respectively. Once these reference thresholds are set, each patient image can be normalized and then binned based on the reference. The defect and low percentages can then be computed. The median thresholding method functions differently: There are no reference‐based thresholds. Each patient image is directly thresholded; all voxels below the 70% of the median of the current image are classified as defect.

The normalization cut‐off proposed in the original paper for HP ^129^Xe MRI maps is the 99th percentile of the currently analyzed map. Our first step was to derive normalization cut‐offs based on the distribution of MP‐MRI maps. A grid search was run, aiming at a cut‐off both leading to an approximate Gaussian curve and optimizing the discriminability of the linear binning, based on the distribution parameters of the reference histogram (mean, standard deviation, median, peak, skewness) and based on AUCs from the ROC analysis conducted on the validation dataset. The original cut‐off, the 99th percentile, was compared to several others including the generalized linear binning method by the same author [28] (using a Box‐cox transformation) and a robust outlier detection method; the median plus X times the median absolute deviation (MAD) [40, 41]. The detailed grid search can be found in the Supporting Information.

Once each ventilation‐weighted and perfusion‐weighted map was normalized, its signal intensities were compiled into a respective histogram. The normalized histograms were binned around their peaks, based on the assumption that most of the lungs should be normally ventilated (or perfused) in healthy children. The exact mathematical definitions for each bin were: normal (peak ±1 SD), high (> peak +1 SD), low (between peak −2 SD and −1 SD), and defect (< peak −2 SD).

Once the healthy reference thresholds defining the bins were computed, both perfusion‐weighted and ventilation‐weighted maps of the subsequent datasets were binned accordingly; each slice was first normalized with the appropriate cut‐off and then binned based on the reference thresholds.

The primary outcomes of the linear binning were the defect percentage and the low‐function percentage. These were calculated by dividing the number of voxels in the respective bin (defect or low) by the total number of lung voxels, then multiplying by 100. For the median‐based method, the standard defect percentage was calculated. For each map, the sum of all voxels under 70% of the median was divided by the total number of voxels and multiplied by 100. Outcomes were abbreviated for perfusion and ventilation, respectively. Linear binning outcomes were the defect percentage (QDP_B_, VDP_B_) and the low‐function percentage (LQP, LVP). The median‐based outcome was the defect percentage (QDP_M_, VDP_M_).

### 2.4 Linear Binning—Validation

To validate the linear binning method, the discrimination between the healthy controls and children with CF for all outcomes (the QDP_B_, LQP and the VDP_B_, LVP) was statistically assessed with a left‐sided Welch's *t*‐test with a Bonferroni‐corrected alpha level 0.013, assuming unequal variances and that children with CF will have higher outcomes. Due to the one‐tailed nature of the tests, the upper bounds of the confidence intervals extend to infinity. An a priori power analysis with an alpha of 0.05, a power of 0.90, a ratio CF/HC of 0.73 and an effect size of 0.8 was conducted. The effect size was based on the PREFUL study comparing the linear binning VDP between healthy adults and adults with CF [35]. The minimum required sample size was 34 HC and 24 children with CF. Our dataset is therefore sufficient for our analysis.

To quantitatively analyze the discriminatory power of the adapted linear binning, ROC curves were generated and their area under the curve (AUC) was extracted. The discriminative power was deemed acceptable if the AUCs were between 0.7 and 0.8, and excellent if between 0.8 and 0.9 [42]. These outcomes were then validated against the AUCs of the standard 70% median threshold.

### 2.5 Linear Binning—Application

A case analysis of two patients is presented to illustrate the complementary value of the linear binning. The two children used in this case are pre‐ETI treatment. They were chosen to exemplify scenarios where the median‐based threshold and the reference‐based linear binning yield divergent classifications of lung function. Only ventilation‐weighted maps are shown, as perfusion‐weighted maps follow the same pattern.

To monitor the longitudinal changes in lung perfusion (ventilation) during the ETI treatment of children with CF, all outcomes pre‐ and post‐start of therapy were tested with a right‐sided paired *t*‐test with a Bonferroni‐corrected alpha of 0.01. As the effect size was difficult to estimate a priori, a post hoc sensitivity power analysis was performed. Using alpha 0.05, a power of 80% and our sample size of 24, a minimum effect size of 0.52 was calculated, meaning this test would detect 80% of changes above this effect size. The resulting effect size was computed using Glass' delta [43], noted *Δ*. An individual case pre‐ and post‐ therapy is also displayed to illustrate the group‐level results.

All analyses were performed using MATLAB (R2022a, The MathWorks Inc., Natick, Massachusetts), G*Power (3.1) [44] and Python (version 3.9, Python Software Foundation).

## 3 Results

### 3.1 Study Population

The children in the reference dataset were on average 14.3 years (SD = 5.3 years), and 52.6% were girls. The children in the validation dataset were on average 13.8 years (±5.5, 46% of girls) for healthy children and 12.5 years (±3.2, 66.7%) for children with CF. Lastly, the 24 children under ETI treatment were on average 13 years (±2.27) before the start of the therapy, and 14.1 years (±2.30) at the second MRI, with 41.2% of girls.

### 3.2 Linear Binning—Reference

The results of the grid‐search for the normalization cut‐offs are detailed in the Supporting Information. The final cut‐offs were chosen based on the distribution of the reference histogram and on the AUCs from the ROC curve analysis of HC and children with CF from the validation dataset, see Table S1 and Figures S2–S5. The final normalization cut‐off for perfusion‐weighted maps was the median plus 4 times the MAD and the median plus 5 times the MAD for ventilation‐weighted maps.

All the ventilation‐ and perfusion‐weighted maps were normalized with their respective cut‐off and stacked into a reference distribution. The perfusion reference distribution, plotted in white in Figure 2A, had a peak at 0.48, a mean of 0.54 and a standard deviation of 0.18. The healthy reference thresholds for the perfusion bins were: < 0.12 for defect, 0.12–0.30 for low, 0.30–0.66 for normal and > 0.66 for high. The ventilation reference distribution, in white in Figure 2C, had a peak at 0.48, a mean of 0.48 and a standard deviation of 0.17. The healthy reference thresholds for the ventilation bins were: < 0.15 for defect, 0.15–0.32 for low, 0.48–0.65 for normal and > 0.82 for high.

**FIGURE 2:**
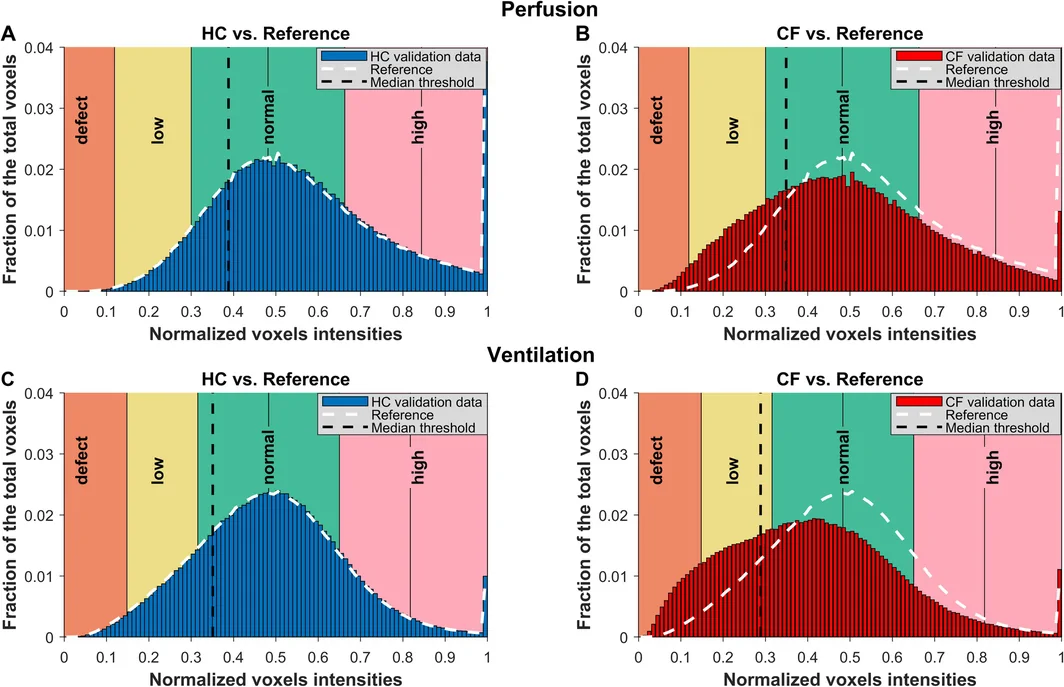
Comparison of the validation dataset against the healthy reference for both perfusion and ventilation. The bins defined from the healthy reference dataset (defect, low, normal and high) are displayed on all panels, along with the shape of the healthy reference histogram in dotted white and the average of the standard threshold of 70% of the median in dotted black. (Panels A and C) show the intensity histogram of the HC of the validation dataset in blue against the healthy reference, for perfusion and ventilation respectively. (Panels B and D) display the intensity histogram of the children with CF from the validation dataset in red against the healthy reference. All of the histograms represented have 100 bars with a 0.01 width.

### 3.3 Linear Binning—Validation

The validation dataset, comprising HC and children with CF, was binned with the aforementioned healthy reference thresholds. Figure 2 shows a comparison of the normalized histograms of HC and children with CF to the reference one. For both perfusion and ventilation, the averaged intensity histogram of HC was similar to the reference one (Figure 2A,C) with a mean of 0.54 and a standard deviation of 0.18 for perfusion, and a mean of 0.48 and a standard deviation of 0.17 for ventilation.

The normalized histograms of children with CF, depicted in red in Figure 2B for perfusion and in Figure 2D for ventilation, are shifted to the left compared to the healthy reference. The averaged CF intensity histogram had a higher percentage of voxels in the defect bin compared to the HC one (1.4% vs. 0.2% for perfusion, 9.5% vs. 1.5% for ventilation) as well as a higher percentage of voxels in the low bin (18.1% vs. 7.9% for perfusion, 25.6% vs. 14.8% for ventilation) (see Table S2 for more details).

When overlaid on the normalized pooled histograms, the averaged 70% median threshold for healthy controls fell within the normal bin defined by the linear binning, whereas for children with CF, in the ventilation‐weighted maps, it shifted into the low bin, as shown in Figure 2. This highlights the intra‐individual nature of the median approach.

To statistically assess the difference between HC and children with CF, the defect percentage and the low‐function percentages were computed. Children with CF had a significantly higher QDP_B_ compared to HC (mean difference [95% CI]: −1.21 [−Inf, −0.70], *p* < 0.0001) as well as higher LQP (−9.45 [−Inf, −6.84], *p* < 0.0001), see Figure 3A and Table 1 for details. The AUCs for QDP_B_ and LQP were 0.88 and 0.85 respectively (both *p* < 0.0001), while the AUC of the standard median thresholding was 0.89, see Figure 3B.

**FIGURE 3:**
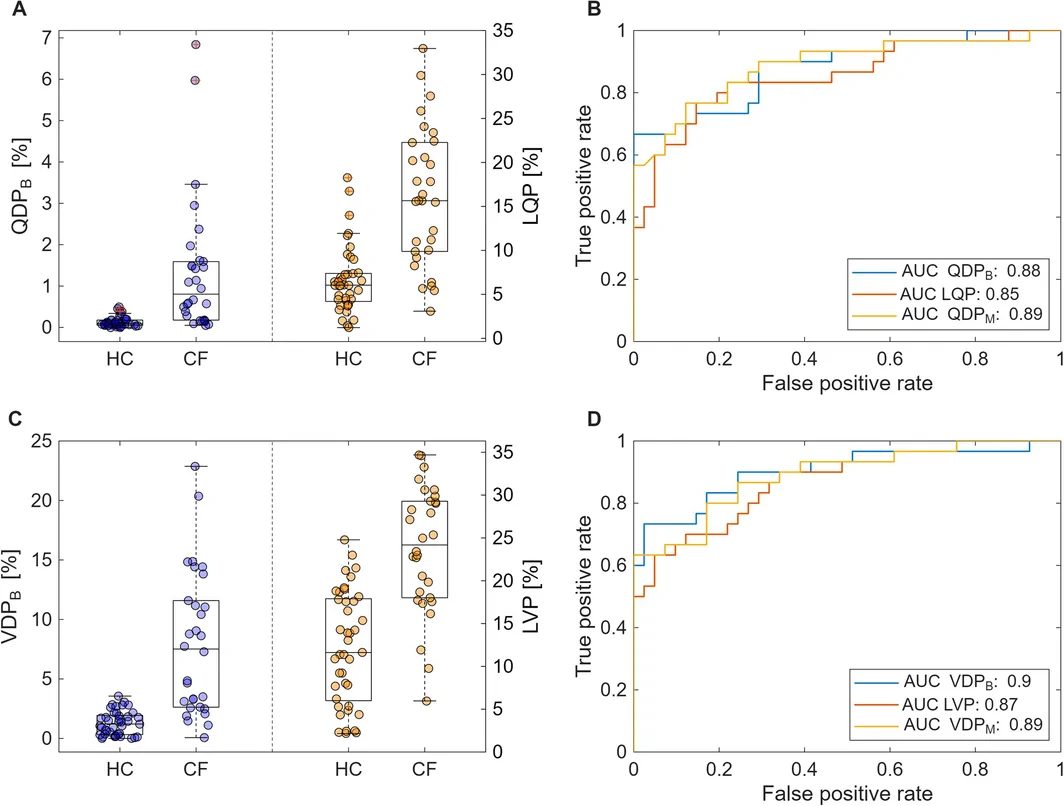
Validation of the discriminative power of our adapted linear binning method. (Panel A) displays the boxplots comparing HC and children with CF for QDP_B_ and LQP respectively, while (Panel C) shows the same for ventilation. (Panels B and D) plot the ROC curves of the binning outcomes, as well as the defect percentage obtained with the median thresholding, for perfusion and ventilation with their respective AUCs.

**TABLE 1:** Outcomes from both the linear binning and the median‐based method along with the statistical test results from the validation and the treatment effects.

	VDP_B_	LVP	VDP_M_	QDP_B_	LQP	QDP_M_
**Validation**						
HC (SD), N = 41	1.18 (0.92)	11.23 (5.90)	15.59 (4.49)	0.20 (0.18)	9.37 (5.27)	14.06 (2.86)
CF (SD), N = 30	7.05 (5.44)	21.17 (6.41)	24.18 (5.37)	1.82 (1.93)	18.82 (8.10)	20.81 (4.82)
Mean difference (CI)	−6.66 [−Inf, −4.78]	−11.57 [−Inf, −8.74]	−8.59 [−Inf, −6.58]	−1.21 [−Inf, −0.70]	−9.45 [−Inf, −6.84]	−6.76 [−Inf, −5.10]
*p* values (Cliff's delta, *δ*)	*p* < 0.0001 (*δ* = 0.80)	*p* < 0.0001 (*δ* = 0.74)	*p* < 0.0001 (*δ* = 0.78)	*p* < 0.0001 (*δ* = 0.76)	*p* < 0.0001 (*δ* = 0.71)	*p* < 0.0001 (*δ* = 0.77)
**Treatment effects**						
Pre‐ETI (SD), N = 24	9.17 (7.45)	21.75 (9.11)	24.21 (7.01)	1.95 (2.42)	17.14 (9.04)	21.64 (5.72)
Post‐ETI (SD), N = 24	3.49 (3.56)	15.90 (8.01)	18.88 (5.22)	1.53 (2.29)	13.03 (8.10)	19.00 (5.80)
Relative difference [%]	−9.03	−11.93	−17.06	−0.29	−15.62	−10.66
Mean difference (CI)	5.68 [3.50, Inf]	5.85 [2.73, Inf]	5.34 [3.17, Inf]	0.43 [−0.45, Inf]	4.11 [1.89, Inf]	2.64 [1.15, Inf]
*p* values (Glass delta's, *Δ*)	*p* < 0.0001 (*Δ* = 0.76)	*p* = 0.0019 (*Δ* = 0.64)	*p* < 0.0001 (*Δ* = 0.76)	*p* = 0.21 (*Δ* = 0.18)	*p* = 0.002 (*Δ* = 0.45)	*p* = 0.0029 (*Δ* = 0.46)

Similarly, children with CF had a higher VDP_B_ than HC (−6.66 [−Inf, −4.78], *p* < 0.0001) and a higher LVP (−11.57 [−Inf, −8.74], *p* < 0.0001), as shown in Figure 3C and Table 1. The AUCs for VDP_B_ and LVP were 0.90 and 0.87 respectively (both *p* < 0.0001), and the AUC of the median thresholding was 0.89; see Figure 3D for their ROC curves.

### 3.4 Linear Binning—Application

Two representative patients from the pre‐ETI cohort were selected to illustrate the divergence between the two methods at the individual level, see Figure 4. Both patients (A and B) exhibited similar VDP when calculated with the median thresholding (37.6% and 32.6%, respectively). However, the underlying distributions differed between the two cases.

**FIGURE 4:**
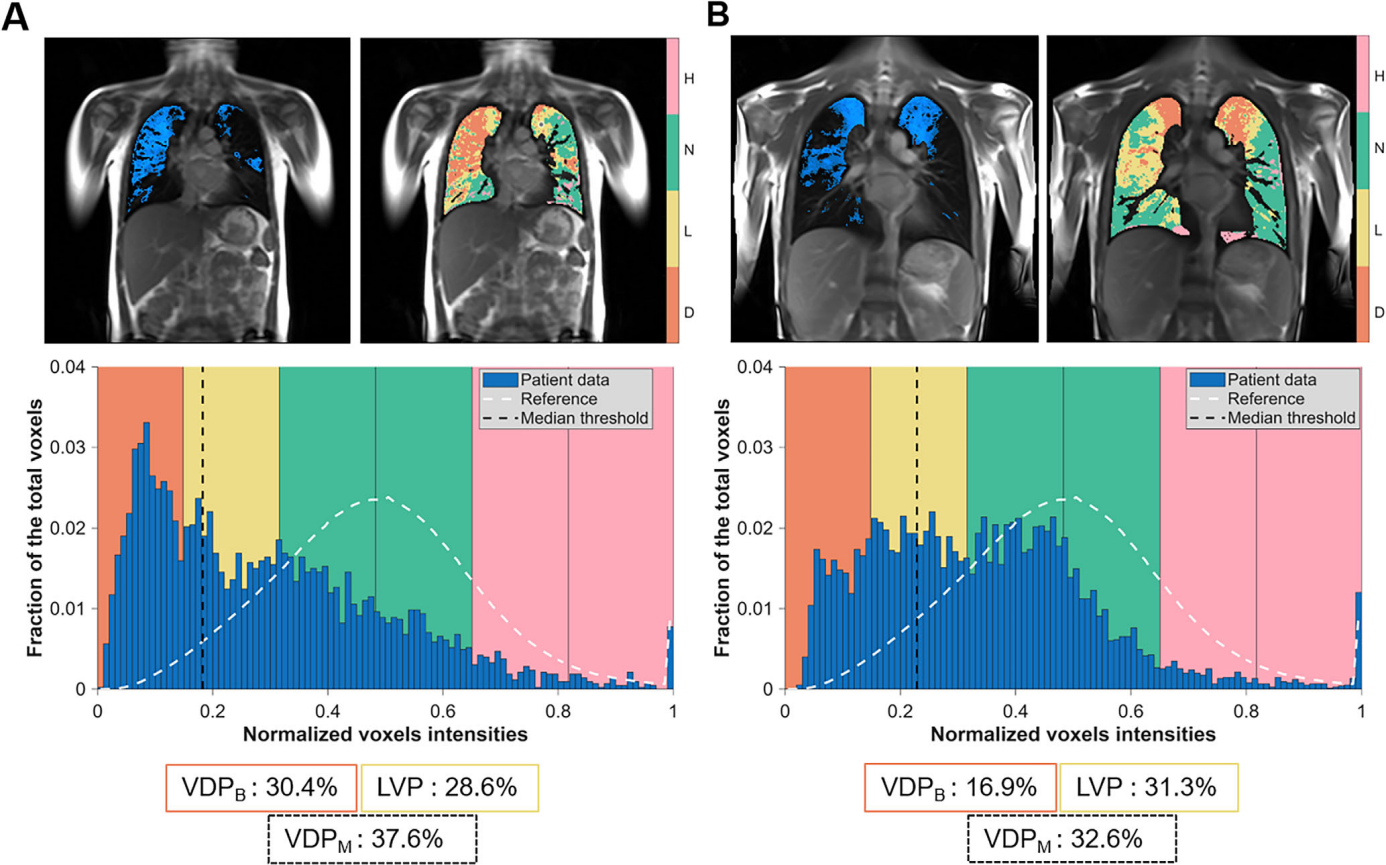
Individual case analysis highlighting the heterogeneity obscured by the standard median thresholding. This figure presents the median‐based thresholding defect map and the linear binning map of a ventilation‐weighted scan of two patients, and their associated distribution maps. The defect map from the median‐based thresholding has a blue label for defect, the rest is 0 (not shown). The categories in the linear binning map have the same color throughout the paper and are labeled “D” for defect, “L” for low, “N” for normal and “H” for high. The distributions are plotted over the linear binning reference bins, and the reference distribution in dotted white. The 70% of the median threshold is represented in dotted black. The linear binning outcomes, the VDP_B_ and LVP, and the median outcome, the VDP, are also shown.

Patient A is a 12‐year‐old girl, with a lung clearance index (LCI) of 13.0. She had a skewed distribution with a peak in the defect bin of the linear binning, see Figure 4A. The linear binning outcomes reflected this, with a VDP_B_ of 30.4% and a LVP of 28.6%. In this case, the sum of the defect and low categories (59%) indicated a broader extent of impairment than standard median thresholding alone. This can be visually inspected in the two defect maps in Figure 4A; the median‐based one on the left and the linear binning one on the right.

Patient B is a 14‐year‐old boy with an LCI of 10.3. He presented a contrasting profile, where the majority of the impaired voxels were in the low bin of the linear binning (LVP = 31.3%, VDP_B_ = 19.9%), see Figure 4A. In this case too, the sum of the linear binning impairment (48.2%) was higher than the median thresholding alone.

To assess the differences between the linear binning and the median‐based thresholding outcomes in monitoring treatment effects, the pre‐ and post‐ETI therapy dataset was binned, and the results are plotted in Figure 5. The mean of the normalized histograms before the start of the ETI treatment was 0.47 (SD: ±0.20) for perfusion and 0.4 (±0.19) for ventilation; the distributions are displayed in Figure 5A,C. After the start of the ETI therapy, a clear shift toward healthier values was visible; see Figure 5B,D. The mean for perfusion was 0.50 (±0.20), and it was 0.46 (±0.18) for ventilation. The median threshold, in dotted black in Figure 5, once again mostly lies in the normal bin, except for one instance, following the intensity distribution of the voxels. For both ventilation and perfusion, the percentage of voxels per bin improved after the start of the therapy; see Table S2 for the full account.

**FIGURE 5:**
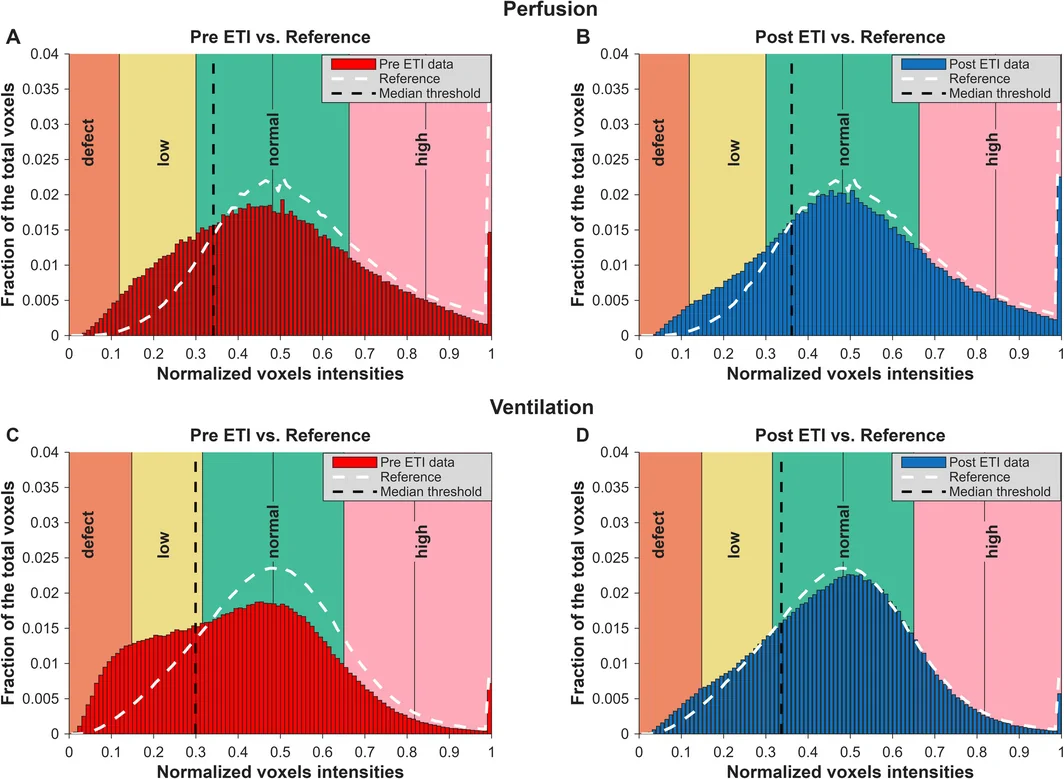
Comparison of the treatment dataset against the healthy reference for both perfusion and ventilation. The bins defined from the healthy reference dataset (defect, low, normal and high) are displayed on all panels, along with the shape of the healthy reference histogram in dotted white and the average of the standard threshold of 70% of the median in dotted black. (Panels A and C) show the intensity histogram at the first time point (“pre,” before the start of the ETI therapy) in red against the healthy reference in dotted white, for perfusion and ventilation respectively. (Panels B and D) display the intensity histogram of the second time point, after the start of therapy (“post”), in blue against the healthy reference in dotted white. All of the histograms represented have 100 bars with a 0.01 width.

Interestingly, the linear binning results showed that while the LQP was significantly reduced after the start of the therapy (4.11 [1.89, Inf], *p* = 0.002, Glass's delta: *Δ* = 0.45), the QDP_B_ was not (0.43 [−0.45, Inf], *p* = 0.21, *Δ* = 0.18), see Figure 6A,B and Table 1. The median thresholding showed the QDP_M_ as significantly decreased after the start of the therapy (QDP_M_, mean difference [95% CI]: 2.64 [1.15, Inf], *p* = 0.0029, *Δ* = 0.46), see Figure 6C and Table 1.

**FIGURE 6:**
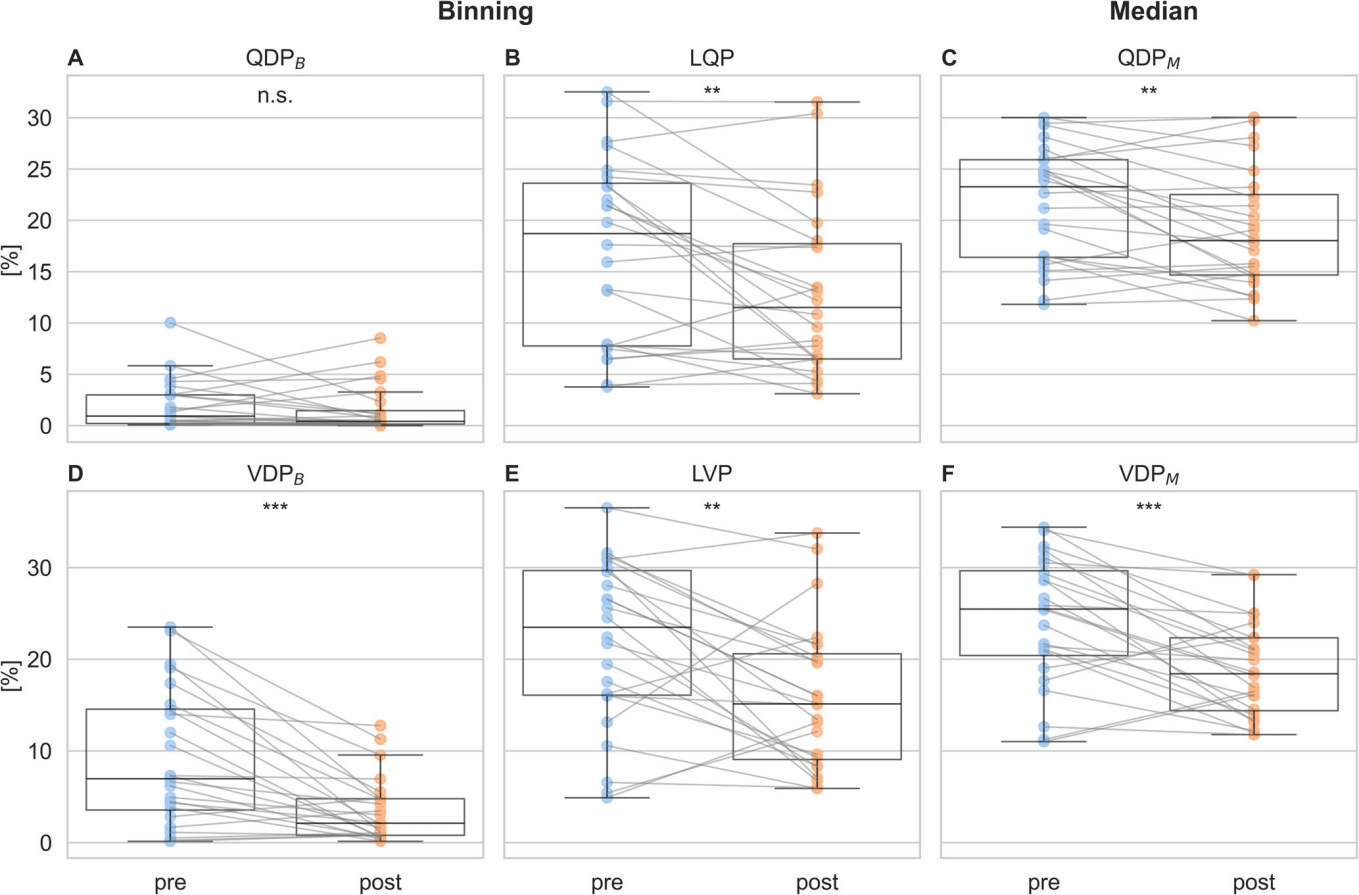
Longitudinal changes between before the start of the ETI therapy, and during. (Panel A and B) display the paired boxplots between the two time points (pre ETI in blue, post ETI in orange) for both the QDP_B_ and LQP. (Panel C) shows the paired boxplots for the QDP_M_, obtained with the median thresholding. (Panels D, E, and F) shows the same but for ventilation. The significance of the statistical tests is displayed with stars.

The ventilation outcomes from the linear binning were all significantly improved post therapy (VDP_B_: 5.68 [3.50, Inf], *p* < 0.0001, *Δ* = 0.76; LVP: 5.85 [2.73, Inf], *p* = 0.0019, *Δ* = 0.64; and VDP_M_: 5.34 [3.17, Inf], *p* < 0.0001, *Δ* = 0.76), see Figure 6D–F and Table 1.

Figure 7 illustrates an example of the complementary information obtained with the linear binning from a patient pre and post ETI therapy. The patient was an 11‐year‐old girl with a LCI of 7.1 at the first time point. She was 12 years old with a LCI of 6.7 at the second time point.

**FIGURE 7:**
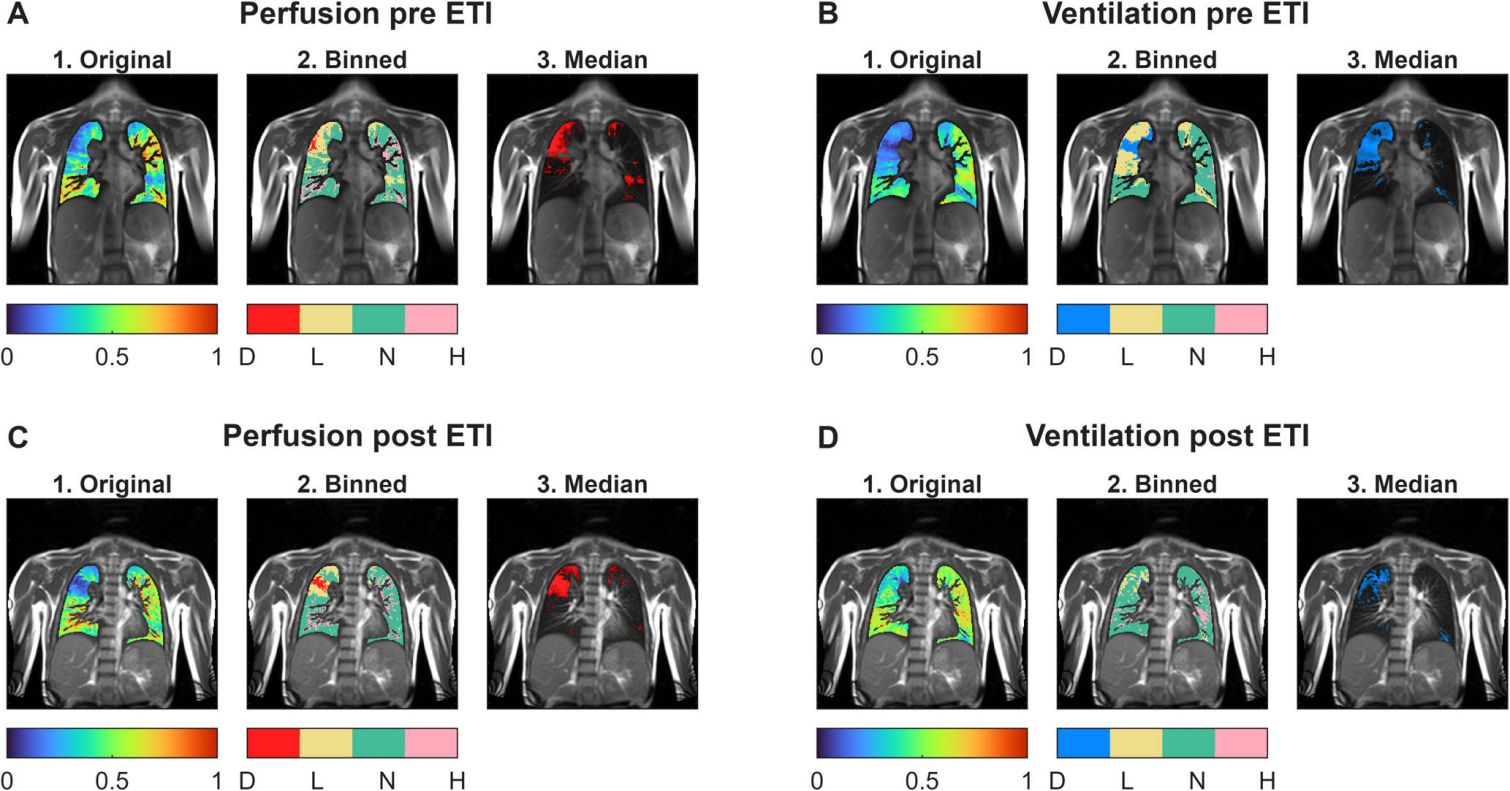
Example of longitudinal changes after the start of therapy for one patient. This figure illustrates the pre and post therapy maps. (Panels A and C) show the following perfusion‐weighted maps pre and post start of ETI therapy respectively: The original maps (1), the binned maps (2) and the maps of defect percentage computed with the median technique (3). (Panels B and D) display the same maps pre and post start of ETI therapy for ventilation. The binning maps have the same colors as on previous figures, except for the defect function which is in red for perfusion and blue for ventilation. The labels are: Defect (D), low (L), normal (N) and high (H).

The perfusion‐weighted maps for this patient showed that the perfusion defect detected by the median technique was similar pre and post therapy (QDP_M_: 24.4% vs. 24.8%), see Figure 7A,C. The binning further characterized these results by revealing a reduction in low perfusion (LQP: 23.53% vs. 15.82%) but a small increase in perfusion defect (QDP_B_: 1.98% vs. 4.46%).

The ventilation‐weighted maps post ETI therapy showed a clear reduction in ventilation defect as detected with the median technique (VDP_M_: 31.1% vs. 17.2%), see Figure 7B,D. The binning maps provided further details; there were almost no ventilationdefect post therapy (VDP_B_: 8.57% vs. 0.74%) and a significant reduction of the low ventilation (LVP: 36.77% vs. 9.35%).

## 4 Discussion

To our knowledge, linear binning has only been applied to ventilation‐weighted images from PREFUL MRI [35, 36] but employing the original linear binning method [35] in one instance and a method with a modified normalization and bin construction [36, 45] for HP‐MRI in another. In this study, the linear binning approach, initially described in HP ^129^Xe MRI, was adapted and validated for use in both, perfusion‐ and ventilation‐weighted MP‐MRI maps. This adapted method demonstrated a discriminative power between healthy controls and CF patients that was comparable to the standard median‐based thresholding, confirming its validity as an alternative approach. Even if there are no metrics to directly compare our results to other published studies, the shift of distribution in children with CF compared to the healthy distribution, as well as the excellent discrimination power aligns with the original paper (comparing healthy and adult asthmatic patients) [27]. Similarly, other HP ^129^Xe MRI studies using linear binning to discriminate between healthy individuals and those with various pulmonary diseases like CF or asthma showed a good discrimination between the two conditions [31, 32].

The adapted linear binning method shows valuable advantages as an alternative approach to threshold‐based methods used to compute the usual defect percentage in pulmonary MRI. The main advantage of the linear binning is its fixed reference‐based categories (defect, low, normal and high), which provide a benchmark for all patients and diseases, allowing a rapid visual comparison to a healthy population. Indeed, as the reference is based on a normal distribution of healthy subjects, the voxel intensities can be interpreted independently of the map's own distribution. In our study, while the pooled median threshold appears to align closely with the boundary between the low and normal categories of the binning, encompassing both defect and low bins, this assumption does not hold for all individual distributions. As illustrated by the individual case analysis in Figure 4, in cases of more severe impairments where voxels are predominantly classified in the defect or low category of the linear binning method, the median threshold shifts downward considerably. In this case, the median threshold fails to capture the low‐intensity voxels. This limitation arises from the mathematical definition of the median thresholding; since the median separates the distribution in two equal parts, the VDP will never reach 50%. The linear binning also brought additional information about the underlying impairment distributions when the VDP calculated from the median thresholding were similar. The linear binning method could therefore be an alternative approach in diseases where the impairments are homogeneously spread or with highly skewed distributions.

Another advantage of the linear binning is its refined granularity, which allowed for a differentiated interpretation of treatment effects in our cohort. In the children receiving ETI therapy, both the standard median threshold method and the linear binning method agreed regarding ventilation: post‐therapy improvements were observed in all outcomes, in line with already published articles [8, 46]. However, while the standard median threshold method indicated a significant improvement in perfusion defects broadly, the linear binning method provided a more specific categorization of this change; only the low‐perfusion category significantly improved after the first few months of therapy, while the perfusion defect category remained unchanged. It is theoretically possible to achieve similar granularity using the median thresholding method; however, this would require extensive, tailored analysis to manage its intra‐individual nature.

The linear binning technique is not the only method categorizing the voxels in different bins. One well‐known technique is the adaptive k‐means algorithm [30]. This algorithm partitions voxels into clusters through two iterations of k‐means clustering. To decide how many starting clusters should exist (four or five), the decision relies on the low‐intensity‐signal percentage. The map is classified into 10 bins, and the percentage of voxels in this first bin represents this low‐intensity‐signal value. Depending on this data‐driven variable, the number of clusters will be chosen, as well as how the defect cluster will be built. There are three different thresholds based on the low‐intensity‐signal percentage value, and in each case, a different number of sub‐clusters will be taken into account to create the defect cluster. The method to compute this low‐intensity‐signal percentage and to define these three thresholds was built from and for HP‐MRI maps. To our knowledge, no study verified that this low‐intensity‐signal percentage and its thresholds were adequate for MP‐MRI or any proton MRI. One cannot be sure that this method would be exactly the same for MP‐MRI, as discussed in previous articles using the PREFUL modality [47]. Additionally, the adaptive k‐means algorithm is highly data‐driven and mathematical, and, in line with the authors of the original linear binning paper, we agree that the methodology is easier to interpret from a physiological standpoint in this context.

One of the strengths of this study is that we adapted the linear binning method specifically to MP‐MRI maps, notably enabling the processing of perfusion maps for the first time, with a similar processing time as the median thresholding. We used a comparably large number of scans from healthy children to build the reference thresholds, 154 scans (and multiple slices per scan) compared to 10 in the original article [27], which is another strength of our study. However, all data originates from a single center, and the lack of standardization of protocols and analysis between centers could impact the results, which has not yet been fully described [36, 48]. It is unclear if the reference thresholds are applicable to data from other centers. Additionally, accounting for age‐dependency in the construction of the reference thresholds might be important [49, 50]. As the reference values in this paper were constructed from pediatric data, they may only be applicable to the pediatric age range. The age‐dependency in the pediatric range has not yet been studied, and it remains unclear if multiple age‐dependent healthy reference thresholds are needed. Future work could examine the need for these age‐dependent reference thresholds.

Additionally, the reference thresholds defined in this paper should be compared to those from other centers performing MP‐MRI analysis, and our adapted method should be compared to the different linear binning methods of other functional lung MRI techniques, as well as other algorithms like the (adaptive) k‐means clustering and to other clinical lung function tests. In the future, the use of the linear binning approach in clinical application should be evaluated.

## 5 Conclusions

We successfully adapted the linear binning method for use in perfusion‐ and ventilation‐weighted MP‐MRI maps. We validated it against the standard median‐based threshold method and showed that it can provide additional information. This innovative approach enhances the assessment of lung function in pediatric populations and offers a framework for further advancements in processing functional lung MRI maps. Future research should explore the applicability of these reference thresholds across different centers as well as age and disease groups, and compare this method to other MRI techniques and lung function tests.

## Funding

This work was supported by Cystische Fibrose Schweiz, Schweizerischer Nationalfonds zur Förderung der Wissenschaftlichen Forschung (10002556, 10004139).

## Conflicts of Interest

Philipp Latzin received grants/contracts from Vertex and OM Pharma, payments or honoraria for lectures/presentations by Vertex, Vifor and OM Pharma, participates on a Data Safety Monitoring Board or Advisory Board from Polyphor, Santhera, Vertex, OM Pharma, Vifor, Allecra, Sanofi Aventis. None of these relationships are in association with the current study. Elisabeth Kieninger reports personal fees from Sanofi‐Aventis and Vertex outside of the submitted work.

## Supporting information


**Figure S1:** Dataset workflow. Each dataset is represented, numbered, and associated to the part of our study. The (^1^) dataset is the healthy BILD cohort that was randomly split between the reference and the HC of the validation dataset. The (^2^) dataset comprises the children with CF from an already published study on the effect of salbutamol on lung function and was used to validate the linear binning technique. The last dataset (^3^) has pre and post ETI therapy MRI data, also already published, and was used to illustrate the difference between the linear binning and the median, and to track the treatment effects on lung MRI.
**Table S1:** Summary of the results per cut‐off and modality. This table describes the results of the curve analysis and the ROC analysis for both ventilation and perfusion, The “Box‐cox” title in the perfusion part refers to the generalized linear binning technique, and the DP and LP are referring to the defect percentage and the low percentage respectively.
**Figure S2:** Visualization of the binning reference distribution of the different cut‐offs for ventilation‐weighted maps. Each row represents a cut‐off; the first row is the original binning method, the second is the median plus four times the MAD, the third is the chosen cut‐off, the median plus five times the MAD, while the fourth row is the median plus six times the MAD. All (Panels A) show the stacked histograms from the HC of the validation dataset, in blue, against the shape of the reference dataset, in dotted white, with the binning bins behind. (Panels B) are the same, but plotting the stacked histograms of the children with CF instead.
**Figure S3:** Visualization of validation step of the different cut‐offs for ventilation‐weighted maps. Each row represents a cut‐off; the first row is the original binning method, the second is the median plus four times the MAD, the third is the chosen cut‐off, the median plus five times the MAD, while the fourth row is the median plus six times the MAD. All (Panels A) show the discrimination between HC and children with CF for the VDP_B_, in blue, and the LVP, in orange. (Panels B) are showing the ROCs of the VDP_B_, the LVP and the median VDP (VDP_M_), along with their respective AUCs.
**Figure S4:** Visualization of the binning reference distribution of the different cut‐offs for perfusion‐weighted maps. Each row represents a different cut‐off; the first row is the original binning method, the second is the generalized method by the same authors, the third is the median plus three times the MAD, the fourth is the chosen cut‐off; the median plus 4 times the MAD, while the fifth is the median plus five times the MAD. All (Panels A) show the stacked histograms from the HC of the validation dataset, in blue, against the shape of the reference dataset, in dotted white, with the binning bins behind. (Panels B) are the same, but plotting the stacked histograms of the children with CF instead.
**Figure S5:** Visualization of validation step of the different cut‐offs for perfusion‐weighted maps. Each row represents a different cut‐off; the first row is the original binning method, the second is the generalized method by the same authors, the third is the median plus three times the MAD, the fourth is the chosen cut‐off; the median plus 4 times the MAD, while the fifth is the median plus five times the MAD. All (Panels A) show the discrimination between HC and children with CF for the QDP_B_, in blue, and the LQP, in orange. (Panels B) are showing the ROCs of the QDP_B_, the LQP and the median QDP (QDP_M_), along with their respective AUCs.
**Table S2:** Percentage of voxels per bin. This table provides the percentage of voxels present in each bin for all the datasets.

## Data Availability

The data that support the findings of this study are available on request from the corresponding author. The data are not publicly available due to privacy or ethical restrictions.
