# Supplementary material for: Adapted Linear Binning Method to Assess Pulmonary Ventilation and Perfusion in Children Using Lung Matrix Pencil Decomposition (MP)‐MRI
Source: Magn Reson Med. 2026 Aug 13;96(6):2708–20. doi: 10.1002/mrm.70544 (PMC13619853; doi:10.1002/mrm.70544)
Supplement: Supplementary file 1 [file MRM-96-2708-s001.docx]

Supporting Information

## **Dataset workflow**

**Figure S1: Dataset workflow. Each dataset is represented, numbered, and associated to the part of our study.** The ^1^ dataset is the healthy BILD cohort that was randomly split between the reference and the HC of the validation dataset. The ^2^ dataset comprises the children with CF from an already published study on the effect of salbutamol on lung function and was used to validate the linear binning technique. The last dataset (^3^) has pre and post ETI therapy MRI data, also already published, and was used to illustrate the difference between the linear binning and the median, and to track the treatment effects on lung MRI.

## **Grid-search optimization of the normalization cut-offs**

### Methods

The original binning method^1^, developed for HP-MRI, uses the 99^th^ percentile as a normalization cut-off; all the maps are divided by this cut-off and every voxel above one is set to one. However, HP-MRI and proton MRI have different signal intensity distributions due to inhaled gas VS imaged protons; histograms of perfusion-weighted maps are strongly right-skewed due to the high signal intensity of the blood vessels while ventilation-weighted histograms have lower signal intensities. The original cut-off from HP-MRI needed to be adapted for both perfusion- and ventilation-weighted images.

A grid search was run to find the optimal cut-off was. The cut-off tested were the one from the original method, using a percentile, and a robust outlier detection method, the median + a certain threshold times the median absolute deviation^2^, since the mean and standard deviation method is based on normal distribution characteristics that we cannot guarantee^3^. For the perfusion-weighted images, strongly right-skewed, the generalized linear binning method from the original authors^4^, including a Box-cox transform, was also tested. For the sake of readability, the ‘median + X*MAD’ was sometimes shortened to ‘X*MAD’.

The criteria to optimize for this grid-search were: (i) the distribution being close to a Gaussian one, with the median, the mean and the peak of the histogram close to each other and around 0.5 with a minimum skewness and (ii) the discriminatory power, based on the AUCs of the ROC analysis computed for our validation dataset comprised of HC and children with CF.

### Results

#### Ventilation-weighted maps

Regarding the first criteria, the mean, the median and the peak of the distribution for the 99^th^ percentile and the 5*MAD cut-offs were the closest to 0.5 with an equal skewness, see more details in Table S1 and their associated graphs in Figure S1. However, these 3 values were equal for the 5*MAD cut-off, and the standard deviation was marginally lower.

For the second criteria, the 5*MAD cut-off has the second best AUCs with 0.90 for the VDP and 0.87 for LVP. The best ones are the ones from the 6*MAD, with 0.91 for VDP and 0.87 for LVP.

When optimizing these two criteria, the chosen threshold was the median + 5*MAD as it both approximated a Gaussian-like distribution and had a very good discrimination between HC and children with CF

**Table S1: Summary of the results per cut-off and modality.** This table describes the results of the curve analysis and the ROC analysis for both ventilation and perfusion, The ‘Box-cox’ title in the perfusion part refers to the generalized linear binning technique, and the DP and LP are referring to the defect percentage and the low percentage respectively.

|  | **Ventilation** | | | | **Perfusion** | | | | |
| --- | --- | --- | --- | --- | --- | --- | --- | --- | --- |
|  | **99th** | **4*MAD** | **5*MAD** | **6*MAD** | **99th** | **Box-cox** | **3*MAD** | **4*MAD** | **5*MAD** |
| **Mean** | 0.50 | 0.53 | 0.48 | 0.44 | 0.51 | / | 0.59 | 0.54 | 0.49 |
| **Std** | 0.18 | 0.18 | 0.17 | 0.16 | 0.19 | / | 0.18 | 0.18 | 0.18 |
| **Median** | 0.49 | 0.53 | 0.48 | 0.43 | 0.49 | / | 0.58 | 0.52 | 0.47 |
| **Peak** | 0.49 | 0.54 | 0.48 | 0.44 | 0.42 | / | 0.56 | 0.48 | 0.43 |
| **Skewness** | 0.14 | 0.00 | 0.14 | 0.24 | 0.43 | / | 0.12 | 0.34 | 0.50 |
| **AUCs (DP/LP)** | 0.88/0.80 | 0.90/0.87 | 0.90/0.87 | 0.91/0.87 | 0.79/0.50 | 0.80/0.68 | 0.89/0.84 | 0.88/0.85 | 0.86/0.86 |

**Figure S2: Visualization of the binning reference distribution of the different cut-offs for ventilation-weighted maps.** Each row represents a cut-off; the first row is the original binning method, the second is the median plus four times the MAD, the third is the chosen cut-off, the median plus five times the MAD, while the fourth row is the median plus six times the MAD. All panels A show the stacked histograms from the HC of the validation dataset, in blue, against the shape of the reference dataset, in dotted white, with the binning bins behind. Panels B are the same, but plotting the stacked histograms of the children with CF instead.

**Figure S3: Visualization of validation step of the different cut-offs for ventilation-weighted maps.** Each row represents a cut-off; the first row is the original binning method, the second is the median plus four times the MAD, the third is the chosen cut-off, the median plus five times the MAD, while the fourth row is the median plus six times the MAD. All panels A show the discrimination between HC and children with CF for the VDP_B_, in blue, and the LVP, in orange. Panels B are showing the ROCs of the VDP_B_, the LVP and the median VDP (VDP_M_), along with their respective AUCs.

#### Perfusion-weighted maps

Regarding the first criteria, all cut-offs except the 3*MAD had a mean and median close the 0.5, see Table S1 and Figure S2. However, the 4*MAD was the only cut-off with a peak close to 0.5 and a lower skewness.

The 4*MAD cut-off also had a better compromise between both the AUC of the QDP (0.88) and the AUC of the LQP (0.85), the other cut-offs having either a higher AUC for the QDP but a lower AUC for the LQP, as the 3*MAD, or lower AUCs in general, see Table S1.

As a result, the cut-offs that optimized our criteria best was the median + 4*MAD.

**Figure S4: Visualization of the binning reference distribution of the different cut-offs for perfusion-weighted maps.** Each row represents a different cut-off; the first row is the original binning method, the second is the generalized method by the same authors, the third is the median plus three times the MAD, the fourth is the chosen cut-off; the median plus 4 times the MAD, while the fifth is the median plus five times the MAD. All panels A show the stacked histograms from the HC of the validation dataset, in blue, against the shape of the reference dataset, in dotted white, with the binning bins behind. Panels B are the same, but plotting the stacked histograms of the children with CF instead.

**Figure S5: Visualization of validation step of the different cut-offs for perfusion-weighted maps.** **.** Each row represents a different cut-off; the first row is the original binning method, the second is the generalized method by the same authors, the third is the median plus three times the MAD, the fourth is the chosen cut-off; the median plus 4 times the MAD, while the fifth is the median plus five times the MAD. All panels A show the discrimination between HC and children with CF for the QDP_B_, in blue, and the LQP, in orange. Panels B are showing the ROCs of the QDP_B_, the LQP and the median QDP (QDP_M_), along with their respective AUCs.

## **Percentage of voxels in each bin of the stacked histograms**

**Table S2: Percentage of voxels per bin**. This table provides the percentage of voxels present in each bin for all the datasets.

|  | **Perfusion [%]** | | | | | **Ventilation [%]** | | | | |
| --- | --- | --- | --- | --- | --- | --- | --- | --- | --- | --- |
|  | **Reference** | **HC** | **CF** | **Pre** | **Post** | **Reference** | **HC** | **CF** | **Pre** | **Post** |
| **Defected** | 0.2 | 0.2 | 1.4 | 2.3 | 1.9 | 1.9 | 1.6 | 9.5 | 10.7 | 4.1 |
| **Low** | 8.2 | 7.9 | 18.1 | 19.0 | 14.7 | 15.3 | 14.8 | 25.6 | 23.9 | 16.9 |
| **Normal** | 67.9 | 67.8 | 60.7 | 59.6 | 62.8 | 67.8 | 68.0 | 54.6 | 55.0 | 65.1 |
| **Elevated** | 23.7 | 24.2 | 19.8 | 19.2 | 20.5 | 15.0 | 15.6 | 10.3 | 10.4 | 13.9 |

# References

1.	He M, Driehuys B, Que LG, Huang YCT. Using Hyperpolarized 129Xe MRI to Quantify the Pulmonary Ventilation Distribution. *Acad Radiol*. 2016;23(12):1521-1531. doi:10.1016/j.acra.2016.07.014

2.	Leys C, Ley C, Klein O, Bernard P, Licata L. Detecting outliers: Do not use standard deviation around the mean, use absolute deviation around the median. *J Exp Soc Psychol*. 2013;49(4):764-766. doi:https://doi.org/10.1016/j.jesp.2013.03.013

3.	Howell DC, Rogier M, Yzerbyt V, Bestgen Y. Statistical methods in human sciences. *New York Wadsworth*. 1998;721:1998.

4.	He M, Wang Z, Rankine L, et al. Generalized Linear Binning to Compare Hyperpolarized (129)Xe Ventilation Maps Derived from 3D Radial Gas Exchange Versus Dedicated Multislice Gradient Echo MRI. *Acad Radiol*. 2020;27(8):e193-e203. doi:10.1016/j.acra.2019.10.016
